# TETRA: a web-service and a stand-alone program for the analysis and comparison of tetranucleotide usage patterns in DNA sequences

**DOI:** 10.1186/1471-2105-5-163

**Published:** 2004-10-26

**Authors:** Hanno Teeling, Jost Waldmann, Thierry Lombardot, Margarete Bauer, Frank Oliver Glöckner

**Affiliations:** 1 Microbial Genomics Group, Max Planck Institute for Marine Microbiology, D-28359 Bremen, Germany; 2 International University Bremen, D-28759 Bremen, Germany

## Abstract

**Background:**

In the emerging field of environmental genomics, direct cloning and sequencing of genomic fragments from complex microbial communities has proven to be a valuable source of new enzymes, expanding the knowledge of basic biological processes. The central problem of this so called metagenome-approach is that the cloned fragments often lack suitable phylogenetic marker genes, rendering the identification of clones that are likely to originate from the same genome difficult or impossible. In such cases, the analysis of intrinsic DNA-signatures like tetranucleotide frequencies can provide valuable hints on fragment affiliation. With this application in mind, the TETRA web-service and the TETRA stand-alone program have been developed, both of which automate the task of comparative tetranucleotide frequency analysis.

*Availability: *

**Results:**

TETRA provides a statistical analysis of tetranucleotide usage patterns in genomic fragments, either via a web-service or a stand-alone program. With respect to discriminatory power, such an analysis outperforms the assignment of genomic fragments based on the (G+C)-content, which is a widely-used sequence-based measure for assessing fragment relatedness. While the web-service is restricted to the calculation of correlation coefficients between tetranucleotide usage patterns of submitted DNA sequences, the stand-alone program generates a much more detailed output, comprising all raw data and graphical plots. The stand-alone program is controlled via a graphical user interface and can batch-process a multitude of sequences. Furthermore, it comes with pre-computed tetranucleotide usage patterns for 166 prokaryote chromosomes, providing a useful reference dataset and source for data-mining.

**Conclusions:**

Up to now, the analysis of skewed oligonucleotide distributions within DNA sequences is not a commonly used tool within metagenomics. With the TETRA web-service and stand-alone program, the method is now accessible in an easy to use manner for a broad audience. This will hopefully facilitate the interrelation of genomic fragments from metagenome libraries, ultimately leading to new insights into the genetic potentials of yet uncultured microorganisms.

## Background

At present, a majority of the microbes from natural microbial communities cannot be transferred into pure cultures, either because proper cultivation conditions have yet to be found, or due to currently unidentified fundamental obstacles [1]. For decades, this has limited our understanding of the functioning of microbes within their natural habitats, such as their metabolic roles, interactions and dependencies.

The metagenome-approach allows for the first time to circumvent the restrictions imposed by the limited culturability of environmental microorganisms [2]. Now, DNA fragments of 40 – 150 kb of uncultured microorganisms can be cloned directly from the environment. This delivers insights into the microorganisms' genetic potentials, sometimes even allowing the reconstruction of entire genomes. Prominent example include the unexpected finding of bacteriorhodopsin in marine *Gammaproteobacteria *[3-6], and the almost complete reconstruction of two bacterial genomes from an acid mine drainage microbial biofilm [7], respectively.

However, the metagenome-approach is not without its limitations and problems. A major constraint is that especially small genomic fragments, as are obtained from libraries that have been constructed with fosmids or cosmids as cloning vectors, often lack suitable phylogenetic marker genes. This leads to the problem that fragments belonging to the same organism cannot be reliably identified as such unless they overlap. In order to nonetheless interrelate such genomic fragments, measures such as the (G+C)-content or BLAST hits and codon usage of the fragment's coding regions are commonly used to assess whether two unlinked fragments from a metagenome library belong to the same organism. These measures, however, can produce ambiguous or even misleading results, and should be supplemented by additional tools that assess the relatedness of the genomic fragments [8].

Since numerous studies have shown that oligonucleotide frequencies within DNA sequences exhibit species-specific patterns [9-18], comparative analysis of such oligonucleotide frequencies is a promising approach to this problem. For tetranucleotides, it has even been demonstrated that their frequencies carry an innate but weak phylogenetic signal [19]. Comparative analysis of tetranucleotide usage patterns also provides a good balance between computational requirements and attainable resolution. This makes the method particularly well-suited for use as a high-throughput method that can assist in tackling the fragment identification problem in metagenomics [8].

In order to automate and facilitate such an analysis, the TETRA software suite was developed, comprising both, a web-service and a stand-alone program.

## Implementation

The algorithms that are used within TETRA have been described elsewhere [8]. In brief, DNA sequences are extended by their reverse-complements to compensate for different patterns of tetranucleotide over- and underrepresentation between the leading and the lagging strand. Then, the frequencies of all 256 possible tetranucleotides are counted and the corresponding expected frequencies are calculated by means of a maximal-order Markov model from the sequences' di- and trinucleotide composition. In order to evaluate the significance of the level of over- or underrepresentation for each tetranucleotide, the divergence between the observed and expected tetranucleotide frequencies is then transferred into z-scores using an approximation published by Schbath [20,21]. Finally, all DNA sequences are compared in pairs by computing the Pearson's correlation coefficient of their z-scores. Details on the method, its applicability and its limits are given in Teeling *et al. *(2004) and the TETRA online manual.

**Figure 1:**
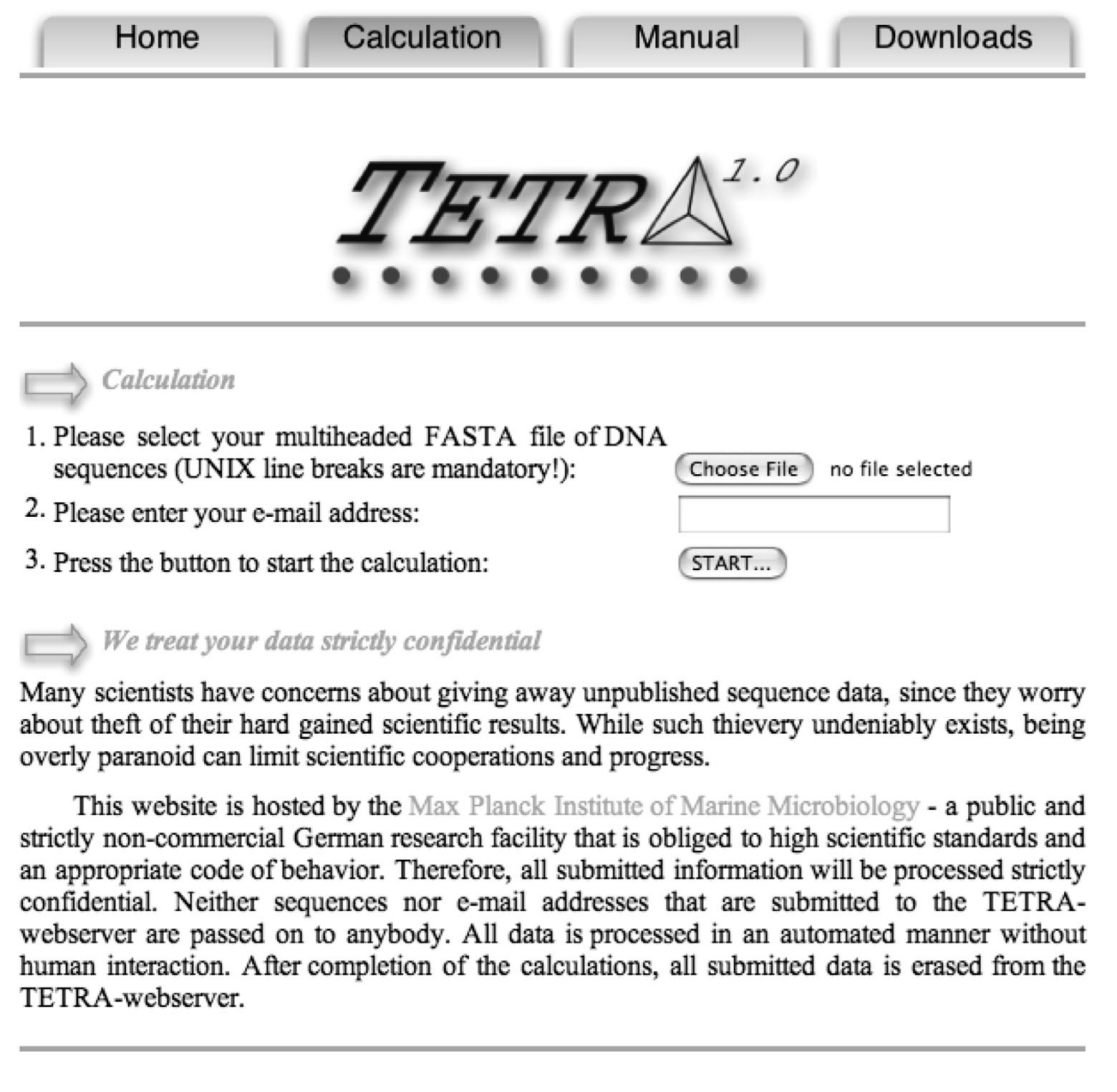
Data-entry page of the TETRA web-service.

The TETRA web-service [22] has been implemented as set of PERL CGI scripts. Access is free to all users. A multi-headed FASTA file with DNA sequence data can be uploaded (actual file size limit: 2 Mb) and after having entered a valid e-mail address, the calculation can be started (Figure 1). Results are sent to the respective e-mail address as a tab-delimited crosstabulation of correlation coefficients in plain text format.

The TETRA stand-alone program can be downloaded for free from the TETRA website [23]. The current release has been implemented in REALbasic ( REAL Software Inc., Austin, Texas) and is available for Mac OS X. Versions for Linux and Windows are also available, but differ in details regarding their implementation and features. The counting of the tetranucleotides in the current version of TETRA stand-alone program is done by ocount – a self-written C program that has been integrated into the program.

## Results and discussion

### TETRA web-service

The TETRA web-service computes correlation coefficients between tetranucleotide usage patterns of DNA sequences, which can be used as an indicator of sequence relatedness. Details on the in- and output formats is available in the comprehensive online documentation [22].

### TETRA stand-alone program

**Figure 2:**
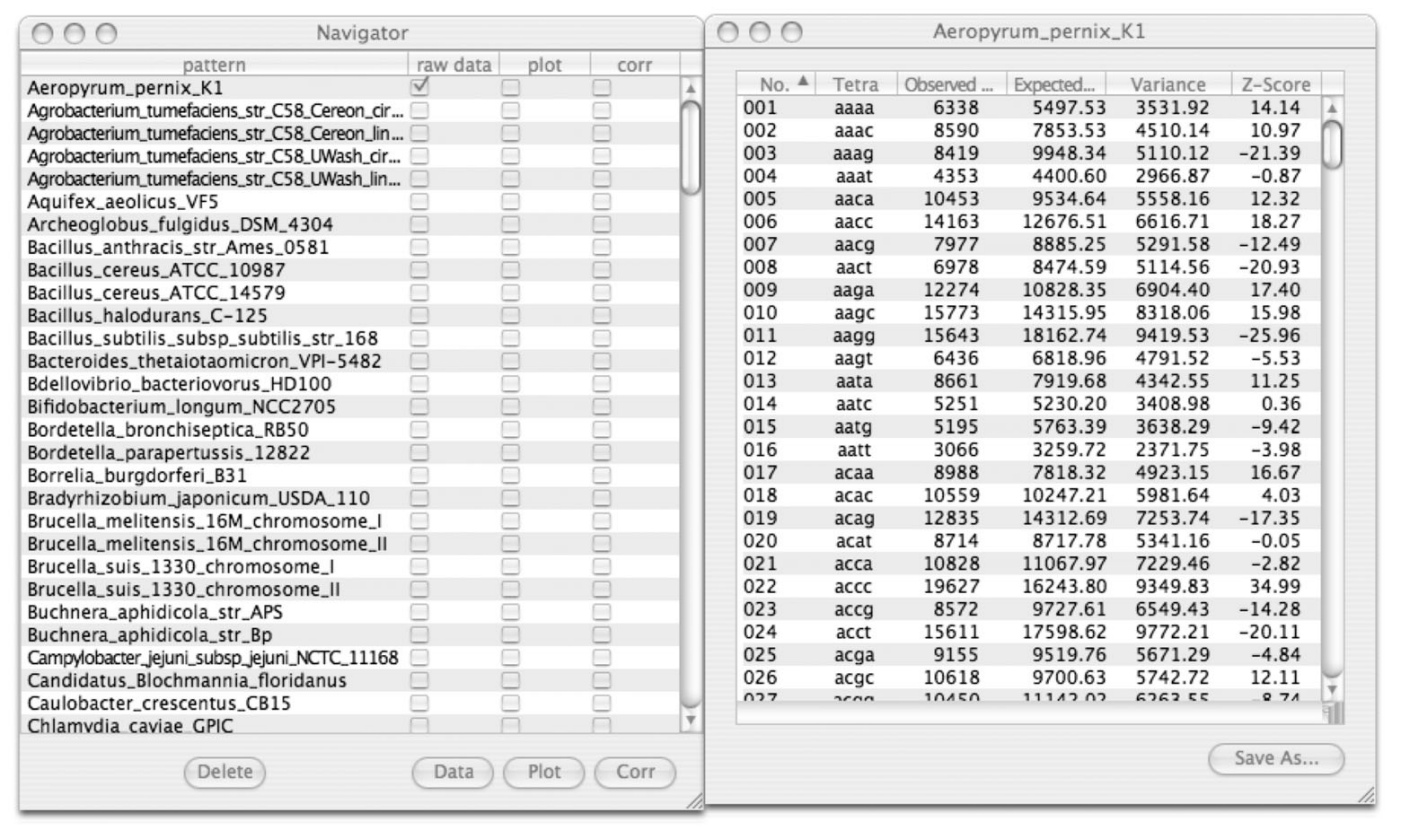
(left) Navigator window of the TETRA stand-alone program showing a subset of the pre-computed 166 prokaryote chromosomes for selection. (right) Data window with a subset of the tetranucleotide frequencies of the *Aeropyrum pernix *K1 chromosome.

The stand-alone version of TETRA has many additional features that are not available via the TETRA web-service. Firstly, it comes with pre-computed tetranucleotide usage patterns of all 166 prokaryote chromosomes that were publicly available by June 2004 (Figure 2). These patterns have been incorporated into the program to provide the user with reference data that can also be used to get familiar with the program. With a few mouse clicks, correlation coefficients for the tetranucleotide usage patterns of all genomes can be computed and exported into PHYLIP format [24]. While not being well-suited for phylogenetic reconstruction, the resolution boundaries of the method can be easily evaluated by looking at the resulting whole genome trees.

**Figure 3:**
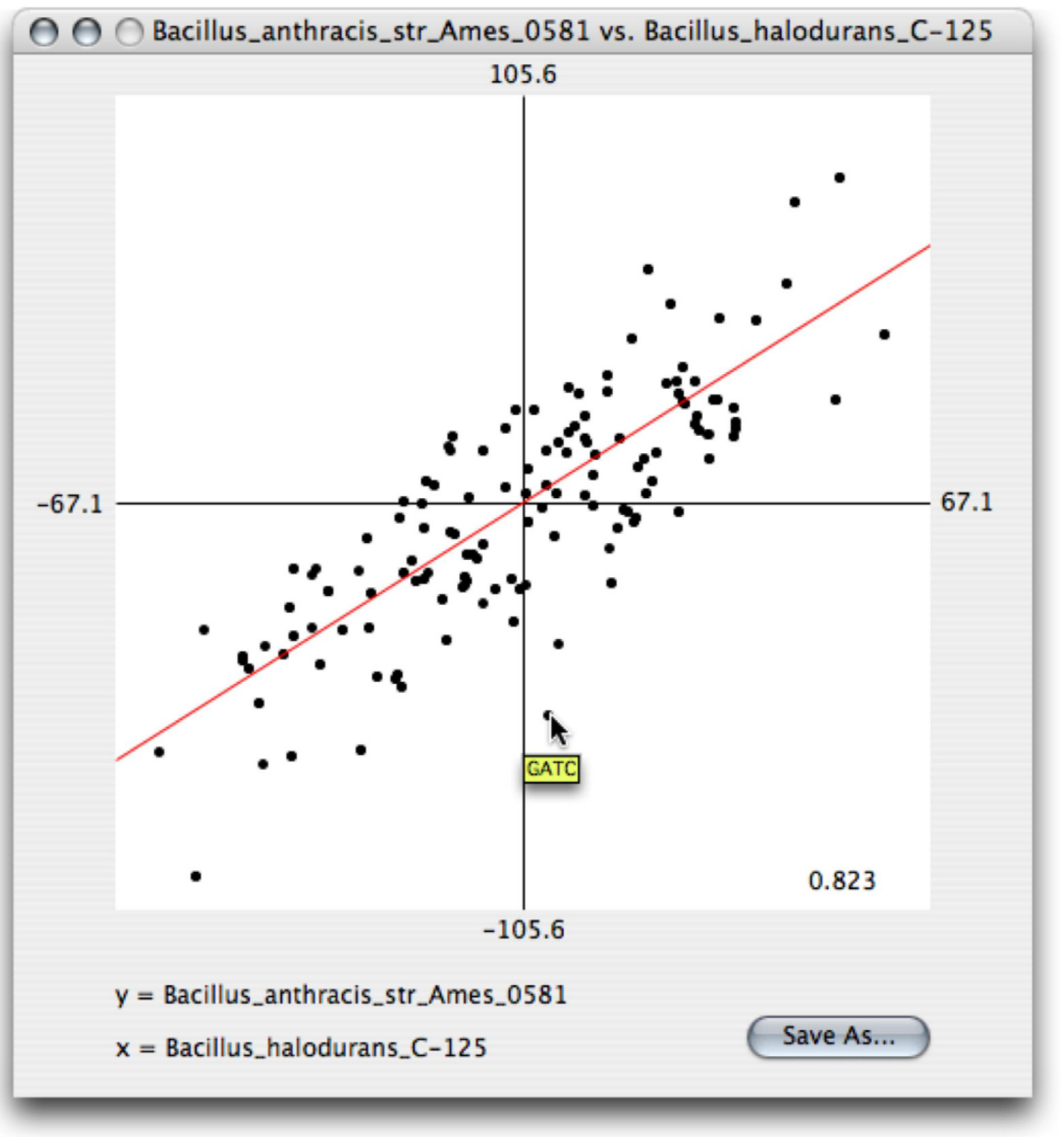
Dotplot of Z-scores for the tetranucleotide frequencies of two *Bacilli*. A regression line is calculated as well as the Pearson correlation coefficient (0.823). Hovering with the mouse over a dot shows a small information window with the tetranucleotide(s) the spot is referring to (as shown for GATC in this case).

Secondly, besides calculating correlation coefficients for tetranucleotide usage patterns, the TETRA stand-alone program allows the user to investigate the raw data (Figure 2) and can produce plots for a more detailed analysis of tetranucleotide over- and underrepresentations (Figure 3). This allows for hints into possible restriction sites by the examination of significantly underrepresented tetranucleotides. Tetranucleotide usage patterns for user-provided sequences can be generated in two ways. Single sequences shorter than 100 kb can be pasted into the so called 'Single Sequence Window'. From there, a sequence can be extended by its reverse complement and its tetranucleotide usage pattern can be calculated. Additionally, the sequence's base composition and GC-content can be computed. Sequences longer than 100 kb or files with multiple sequences can be imported by the 'Batch Mode'. The 'Batch Mode' reads a multi-headed FASTA file and computes the tetranucleotide usage patterns of all sequences within this file in a fully automated manner.

The tetranucleotide usage patterns of an average-sized genome (4 Mb) is computed in less than 10 minutes on a dual 1.8 GHz G5 (IBM PPC 970) computer. Newly computed tetranucleotide usage patterns are displayed within the 'Navigator' window, which is the central place for data management, access to the raw data and the calculation of plots and correlation coefficients (Figure 2). Raw data and correlation coefficients that have been computed for multiple patterns can be saved as tab-delimited tables in plain-text format and the graphical output (2D-plots) can be saved in JPEG-format.

A detailed documentation of the TETRA stand-alone program and its functions is available via the program's online help system.

### Applicability

As has been demonstrated in a previous study [8], the analysis of tetranucleotide usage patterns is often (but not always) a much more reliable measure of sequence relatedness than the (G+C)-content. However, as a sequence-based measure it is affected by local changes in sequence composition. For example, large stretches of horizontally acquired genes will blur the resolution. Likewise, resolution is a function of sequence-length, i.e. the shorter the sequence, the less meaningful a tetranucleotide frequency analysis will be.

While the method works quite well for sequences in the range of 40 kb, it is certainly not suited for the analysis of single-read end-sequences, which are usually shorter than 1 kb. Since the phylogenetic signal within tetranucleotide usage patterns is faint, the method performs weakly for whole genome phylogenetic tree reconstructions. In a whole-genome tree calculated from the pre-computed 166 prokaryotic chromosomes (data not shown), organisms are mostly grouped at the species level and at the level of genera, when these are closely related (i.e. *Escherichia *sp., *Shigella *sp., *Yersinia *sp. or *Mesorhizobium *sp., *Sinorhizobium *sp., *Bradyrhizobium *sp.). However, more distantly related genera or even species with larger evolutionary distances are often not correctly clustered (e.g. *Prochlorococcus *sp.).

Therefore, the analysis of tetranucleotide usage patterns should not be regarded as a tool to deduce phylogenetic relationships, but rather as a fingerprinting technique for genomic fragment correlation. For example, assignment of fosmid-sized genomic fragments from metagenome libraries of a microbial consortia that mediates the anaerobic oxidation of methane was possible using tetranucleotide frequency analysis, and was shown to be in perfect agreement with 16S rRNA sequence analysis [8].

## Conclusions

With the worldwide ongoing programs to sequence and analyze natural communities, new approaches for sequence correlation beyond G+C content, read densities and codon usage have to be developed and made available to the users. The easy to use TETRA software will facilitate this task and provide additional decision support for, e.g., fragment assignment also when complete genomes have to be assembled in environmental sequencing projects.

## Availability and requirements

• Project name: TETRA

• Project home page: 

• Operating system(s): Platform independent (web-service); Mac OS X (stand-alone program)

• Programming language: REALbasic

• Other requirements: none

• License: none

• Any restrictions to use by non-academics: none

## List of abbreviations

BLAST – basic local alignment search tool

megx – marine environmental genomics

## Authors' contributions

TETRA was implemented by HT and JW, TL contributed to the TETRA web-service, MB and FOG contributed important ideas regarding implementation, features and tested the programs.
